# Dermoscopy, Line-Field Confocal Optical Coherence Tomography, Reflectance Confocal Microscopy, and Ultra-High-Frequency Ultrasound: Clues for the Diagnosis of Hidrocystomas

**DOI:** 10.3390/diagnostics14232671

**Published:** 2024-11-27

**Authors:** Maria Erasti, Martina D’Onghia, Anastasia Batsikosta, Mariano Suppa, Linda Tognetti, Simone Cappilli, Ketty Peris, Francesca La Marca, Jean Luc Perrot, Giovanni Rubegni, Pietro Rubegni, Elisa Cinotti

**Affiliations:** 1 Dermatology Unit, Department of Medical, Surgical, and Neurological Sciences, University of Siena, 51300 Siena, Italy; maria.erasti@libero.it (M.E.); francescalamarca93@gmail.com (F.L.M.); pietro.rubegni@unisi.it (P.R.); elisa.cinotti@unisi.it (E.C.); 2 Pathological Anatomy Section, University of Siena, 51300 Siena, Italy; natasha.batsikosta@gmail.com; 3 Department of Dermatology, Hôpital Erasme, HUB, Université Libre de Bruxelles, 1070 Brussels, Belgium; dr.marianosuppa@gmail.com; 4 Department of Dermatology, Sacred Heart Catholic University, 20123 Rome, Italy; simo.cappilli@gmail.com (S.C.); ketty.peris@unicatt.it (K.P.); 5 Department of Dermatology, University Hospital of Saint-Etienne; 42270 Saint-Etienne, France; j.luc.perrot@chu-st-etienne.fr; 6 Ophthalmology Unit, Department of Medicine, Surgery and Neurosciences, University of Siena, 51300 Siena, Italy; giovanni.rubegni@student.unisi.it

**Keywords:** hidrocystomas, dermoscopy, LC-OCT, optical coherence tomography, RCM, UHFUS

## Abstract

Background/Objectives: Hidrocystomas, eccrine and apocrine, are rare cystic lesions that form benign tumors of the sweat glands. This study aimed to describe the clinical features of hidrocystomas and evaluate the role of non-invasive imaging techniques, including dermoscopy, Line-field Confocal Optical Coherence Tomography (LC-OCT), Reflectance Confocal Microscopy (RCM), and Ultra-High-Frequency Ultrasound (UHFUS), in their diagnosis. Methods: In total, seven cases of hidrocystomas were collected from the Dermatologic Clinic of the University of Siena, Italy. Predefined dermoscopic, LC-OCT, RCM, and UHFUS features were retrospectively described. Results: Overall, hidrocystomas were located on the face, mainly presenting as blue/purple-bluish translucent papules (71%). Dermoscopic examination revealed a homogeneous purple-bluish color (71%), white pale halo (71%), and shiny globules at the periphery (57%). LC-OCT identified a hyporeflective cupoliform round structure in the dermis with a bright and thick contour, while UHFUS showed a round structure in the dermis filled with hypoechoic content. Conclusions: Non-invasive imaging techniques could significantly enhance the diagnostic accuracy of hidrocystomas, aid in differentiation from other lesions, and minimize unnecessary biopsies.

## 1. Introduction

Hidrocystomas are rare benign papular or nodular cystic proliferations that often develop in the periorbital area of the face in middle-aged adults [1]. These lesions arise from the apocrine glands of the skin and can manifest as either eccrine or apocrine variants, each with distinct characteristics. Based on histopathological findings, hidrocystomas are classified into apocrine (AHs) and eccrine (EHs) hidrocystomas. AHs are often multilocated and feature apocrine secretion cysts, while EHs are more likely to represent unilocular retention of sweat within a dilated duct or gland, rather than presenting as cystic neoplasms that lack decapitation secretion [2,3].

Clinically, both variants of hidrocystomas present as translucent, skin-colored, or bluish papules or nodules. Upon palpation, these lesions are moderately firm, dome-shaped, and movable, and they are often asymptomatic [4]. Specifically, AHs are generally seen as single papules, whereas EHs can be solitary or, more commonly, present as multiple lesions, often referred to as the Smith and Robinson type [5,6]. It is noteworthy that the presence of multiple hidrocystomas can be associated with rare genodermatoses, including Schöpf–Schulz–Passarge and Goltz–Gorlin syndromes [7].

While clinical presentation plays a crucial role in diagnosing hidrocystomas, dermatologists sometimes encounter challenges in differentiating these lesions from other conditions. These include benign lesions such as epidermoid cysts, dermatofibromas, and blue nevi, as well as malignant tumors like basal cell carcinoma (BCC). BCC is recognized as the most prevalent skin cancer, affecting millions of individuals globally each year, particularly those with fair skin and extensive sun exposure history [8]. Clinically, BCC can mimic hidrocystomas due to their similar appearance and location, which complicates diagnosis. Particularly, BCC presents in various forms, including nodular, superficial, and infiltrative types, often characterized by pearly nodules, scaly patches, or non-healing ulcers [9,10].

Accurate differentiation between hidrocystomas and BCC is fundamental, given that management strategies for these conditions differ significantly. BCC necessitates surgical excision to prevent local invasion, whereas hidrocystomas are benign lesions that are typically managed for cosmetic reasons alone [2]. Therefore, a correct clinical diagnosis may avoid unnecessary skin biopsies that could lead to patient anxiety and additional healthcare costs.

Recently, non-invasive diagnostic imaging techniques, such as dermoscopy, Line-Field Optical Coherence Tomography (LC-OCT), Reflectance Confocal Microscopy (RCM), and Ultra-High-Frequency Ultrasound (UHFUS), have gained more and more attention in clinical practice for the management of cutaneous lesions [11]. These advanced imaging techniques not only improve the accuracy of skin tumor diagnosis but also significantly enhance diagnostic confidence for benign lesions, thereby aiding in the differential diagnosis between malignant and benign conditions [12].

Among these technologies, LC-OCT has demonstrated exceptional performance in real-life clinical practice, particularly in the detection of BCC and in monitoring disease course after medical treatments when surgery is not deemed necessary [13]. Specifically, LC-OCT offers real-time 3D visualization of skin images in both vertical and horizontal views, effectively combining the advantages of optical coherence tomography (OCT) and RCM. This approach overcomes limitations associated with spatial resolution, penetration depth, and image orientation commonly encountered with traditional imaging techniques [14].

To the best of our knowledge, the literature on non-invasive imaging for the diagnosis of hidrocystomas remains limited. Thus, the aim of this study was to describe the dermoscopic, LC-OCT, RCM, and UHFUS features associated with hidrocystomas and to evaluate the potential role of these non-invasive diagnostic techniques in a case series of hidrocystomas.

## 2. Materials and Methods

This retrospective and observational study was conducted between September 2021 and March 2024. The databases of the LC-OCT device (DAMAE Medical, Paris, France) and RCM device (Vivascope 3000^®^, Caliber ID Inc., New York, NY, USA) of the Dermatology Department of the University of Siena were examined. In addition, UHFUS images were also analyzed using the Vevo MD device^®^ (VisualSonics FUJIFILM Inc^®^, Toronto, ON, Canada). A total of seven non-consecutive cases of hidrocystomas were selected, each with at least one acquisition using dermoscopy, LC-OCT, RCM, or UHFUS. Among these cases, histological examination was available for three after surgical excision; in the remaining instances, the diagnosis was based on clinical assessment due to the presence of typical features associated with hidrocystomas. Notably, fluid content was detected upon clinical examination, and these cases exhibited no significant changes after a follow-up period of one year.

Images from the LC-OCT and RCM were acquired and evaluated by two expert dermatologists (E.C. and J.L.P.), both with over ten years of experience in non-invasive imaging techniques. Additionally, the evaluations were analyzed by three dermatologists (M.E., M.D., and F.L.M.) with limited experience in this area (minimum of four months).

The LC-OCT system comprises a portable probe interfaced with a central unit and display, facilitating a rapid acquisition rate of 10 frames per second. It has an axial resolution of 1.2 μm, a lateral resolution of 1.3 μm, a scanning depth of up to 500 μm, and a lateral field of view spanning 1.2 mm. To maintain optimal imaging conditions during the acquisition process, a drop of paraffin oil was applied between the lesion and the camera lens of the LC-OCT system, achieving effective refractive index matching. The system captures vertical sections and/or three-dimensional images that provide critical insights into the anatomical characteristics of the lesions.

Reflectance Confocal Microscopy images measuring 1 mm × 1 mm were obtained using the Vivascope 3000^®^ (Caliber ID Inc., New York, NY, USA) at a selected depth of less than 300 μm. The in vivo RCM examination was performed with a handheld VivaScope 3000 camera (MA-VIG GmbH, Munich, Germany), which utilizes an 830 nm wavelength laser, providing high-resolution images with a lateral resolution of 1 μm and an axial resolution of 3−5 μm. This configuration corresponds to a horizontal section of the skin measuring 920 μm × 920 μm at depths of up to 250 μm. Furthermore, a digital camera (Vivacam, Lucid Inc., Menlo Park, CA, USA) connected to the RCM computer system was employed to capture dermoscopic images while the device was operating in RCM mode.

The sonographic examination was conducted by an expert utilizing the Vevo MD device^®^ (VisualSonics FUJIFILM Inc^®^). This device employs Ultra-High-Frequency Ultrasound (20–100 MHz), which facilitates superior image resolution despite its limited tissue penetration capabilities.

According to the current literature, various dermoscopic patterns were assessed under polarized dermoscopy, including the presence of purple-bluish coloration, white pale halos, whitish globules, and distinct vascular patterns such as arborizing or linear arrangements [10,15]. Similarly, RCM criteria were chosen based on previously described features that are suggestive of hidrocystomas [16,17,18,19]. Finally, the images obtained from LC-OCT and UHFUS were described using terminology proposed in the literature [11,20]. Descriptive statistics were employed to include the frequency and percentage of the features analyzed, thereby providing a comprehensive overview of the findings.

## 3. Results

In total, seven hidrocystomas were included in the study. Overall, three subjects were male, and the age ranged from a minimum of 42 to a maximum of 90 years. The clinical, dermoscopic, and LC-OCT features of the evaluated hidrocystomas are presented in Table 1.

All hidrocystomas were located on the face. Specifically, three lesions were on the eyelid, three were on the forehead, and one was on the cheek. Clinically, most hidrocystomas appeared as purple-bluish translucent papules with a dome-shaped profile (71%), while two pinkish papules were observed. Considering all seven lesions, the dermoscopic examination showed a homogeneous purple-bluish color in five cases (71%), surrounded by a white pale halo. Whitish and shiny globules were observed in four lesions (57%), particularly at the periphery, while chrysalis structures were detected in three (43%) lesions. As regards the vascular pattern, it was arborizing in four cases (42.8%), followed by a linear pattern in two lesions (28%), while one hidrocystoma showed no visible vessels on dermoscopy. LC-OCT images were available in five cases. LC-COCT evaluation revealed hidrocystomas as hyporeflective cupoliform round structures in the dermis with a bright and thick contour. The content of the dark cavity was hyporeflective in four cases, while in one case it was hyperreflective. RCM was performed on two cases, but no signs of hidrocystomas were identified, likely due to their deeper location. Finally, UHFUS was examined in two cases, revealing a round structure in the dermis filled with hypoechoic content, surrounded by a thick superficial roof, a lateral hypoechoic band, and a hyperechoic deep margin, likely due to the fluid content inside these structures.

## 4. Discussion

Hidrocystomas are benign cystic tumors that develop from dermal sweat glands, typically appearing on the face of adults [21,22]. However, clinical diagnosis can be particularly challenging due to their ability to mimic a variety of conditions. Not only can they resemble other benign lesions, such as blue nevi, dermatofibromas, and syringomas, but they can also present similarly to malignant tumors, particularly BCC. This potential for misdiagnosis may lead to unnecessary surgical excision, underscoring the need for accurate diagnostic approaches [17]. In this context, non-invasive imaging techniques, including dermoscopy, LC-OCT, RCM, and UHFUS, could serve as valuable tools for the diagnosis of hidrocystomas [23,24].

In our study, we included seven cases of hidrocystomas located on the face. In three patients, the diagnosis was confirmed histologically. Clinically, the lesions appeared as purple-bluish or pinkish, translucent, oval or ellipsoid, subcentimetric papules characterized by a dome-shaped silhouette, which aligns with previously reported descriptions in the existing literature [10].

### 4.1. Dermoscopy

**Figure 1:**
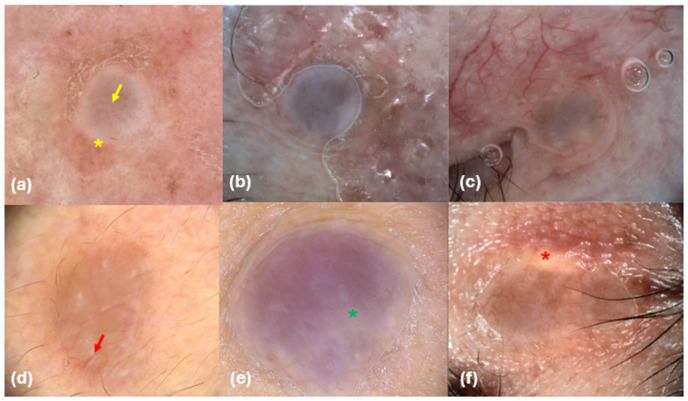
Dermoscopic images of hidrocystomas (**a**–**f**). (**a**) White pale halo (yellow asterisk) and linear vessels (yellow arrow); (**b**) purple-bluish color; (**d**) arborizing vessels (red arrow); (**e**) chrysalis (green asterisk); (**f**) whitish globules at the periphery (red asterisk).

The role of dermoscopy in the clinical diagnosis of adnexal tumors, including hidrocystomas, is well recognized [10,25]. Interestingly, dermoscopy is not only valuable for diagnosing hidrocystomas but can also be employed to monitor the recurrence of EHs following treatment with Botulinum Toxin type A, as suggested by Correia et al. [23]. In our study, five hidrocystomas exhibited a homogeneous purple-bluish color, which corresponds with the typical pigmentation seen in these lesions, ranging from purple-blue to gray-black, as well as shades of yellow and pink [26] (Figure 1). 

This purple-bluish pigmentation likely results from the accumulation of substances such as lipofuscin, melanin, or iron, or may be attributed to the Tyndall phenomenon. However, this characteristic can complicate the diagnosis, as similar pigmentation can also be present in a variety of other conditions, including melanocytic lesions, pigmented BCC, trichoepitheliomas, trichilemmal cysts, syringomas, and even comedones [4].

In our findings, 71% of the lesions were surrounded by a pale white halo, a feature previously noted by Duman et al. in their dermoscopic evaluation of EHs [15]. Additionally, we observed whitish globules in four hidrocystomas (57%), which may represent an incomplete halo due to collagen compression within the cyst. The central globules could correspond to fluid-filled spaces. Conversely, Wang et al. [24] suggested that these white dots could indicate openings of adnexal structures often blocked by keratin and arranged in rosettes. We identified chrysalis structures in three cases. These white linear formations may arise from changes in the orientation of dermal collagen fibers due to the pressure exerted by the tumor. However, similar structures can also be found in melanomas, BCC, Spitz–Reed nevi, and dermatofibromas [27].

In terms of vascular patterns, linear vessels were observed in two lesions (28%), while arborizing vessels were noted in four lesions; one hidrocystoma exhibited no visible vessels. Arborizing vessels are particularly characteristic of the AH subtype but are also a hallmark feature of BCC [28,29]. Indeed, BCC dermoscopy commonly reveals features such as shiny white structures, peripheral blue-gray ovoid nests, and a prominent vascular pattern characterized by arborizing and linear vessels, particularly in its nodular subtype, which serve as key diagnostic criteria for this malignancy [30]. Eccrine hidrocystomas, conversely, have been described as possessing a central convex crater above a defined vessel-free cystic lesion [23,26].

On this basis, while dermoscopy is indeed a valuable non-invasive tool for dermatologists, it may not be entirely sufficient on its own for accurately diagnosing hidrocystomas, especially given the overlap with malignancies like BCC and melanoma [4,10]. The integration of multiple non-invasive imaging techniques may enhance diagnostic accuracy and facilitate better clinical management, allowing for differentiation between benign and malignant lesions.

### 4.2. LC-OCT

**Figure 2:**
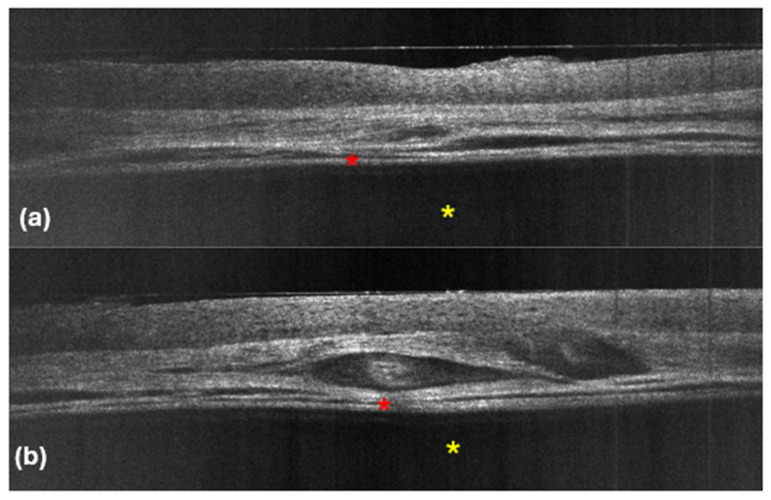
LC-OCT images (**a**,**b**). Vertical sections showing hyporeflective cupoliform area, well defined by a bright and thick upper contour (red asterisks), filled with a completely hyporeflective material (yellow asterisks).

To the best of our knowledge, Verzì et al. [11] were the first to describe the LC-OCT features of five hidrocystomas, which showed a normal epidermis and a dermal cupoliform hyporeflective structure with a thick, hyperreflective border surrounding the dark content. In our study, all five lesions exhibited a similar hyporeflective cupoliform area, which was well defined by a bright and thick upper contour, corresponding to the top of the hidrocystoma (Figure 2).

Regarding the content of the dermal structure, 80% of the lesions were filled with a completely homogeneous hyporeflective material, indicating a liquid nature, which is consistent with the fluid content typically found in hidrocystomas. Interestingly, one lesion exhibited a slight hyperreflective substance within a darker region, suggesting that this material may contain elements such as lipofuscin or other debris that can contribute to the varying echogenicity observed in these tumors [20]. The hyporeflectivity is likely indicative of liquid, while hyperreflectivity can suggest the presence of denser substances.

In terms of vascular structures, they were not consistently visible across all cases. Some LC-OCT images revealed dilated vessels situated above the cupoliform area, which may reflect the vascular supply typical of these lesions. A thick hyperreflective contour surrounded the cupoliform structure, and in certain instances, a cleft was observed between this contour and the adjacent dermis, possibly indicating a delineation between the cystic lesion and surrounding tissues. Notably, we did not detect any architectural abnormalities or the presence of small bright cells in the epidermis overlying the hidrocystoma, which is a crucial finding.

In contrast, the presence of specific features in BCC, as detailed by Suppa et al. [31], highlights the diagnostic significance of LC-OCT in differentiating these two entities (Table 2).

**Figure 3:**
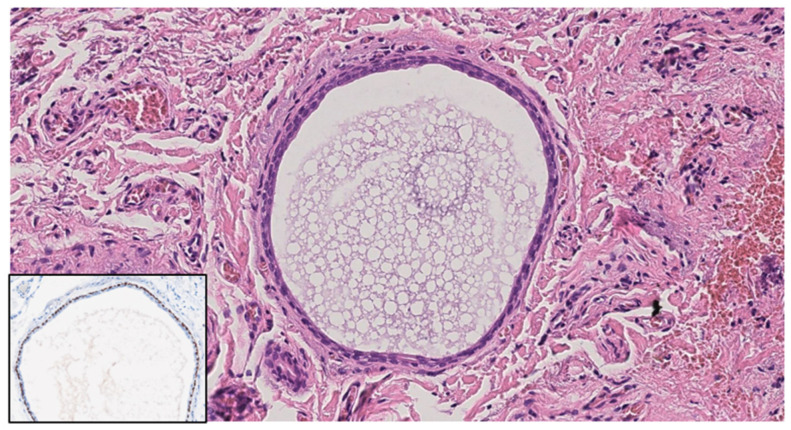
Histological image. Dermal cyst is composed of an inner layer of cuboidal epithelium and an outer myoepithelial cell layer; p63 (insert) highlights myoepithelial cells. Hematoxylin and Eosin; original magnification (OM) ×20; immunohistochemistry, chromogen diaminobenzidine (insert); original magnification (OM): p63, ×20.

Basal cell carcinoma typically presents with distinct architectural abnormalities, branched lobules with a millefeuille pattern, clefting, and a darker rim. These characteristics are essential for the diagnosis of BCC and were absent in our hidrocystoma cases, reinforcing the benign nature of these lesions. Additionally, the lack of such features emphasizes the importance of LC-OCT in identifying the unique characteristics of hidrocystomas, thereby aiding in their differentiation from BCC. Thus, by employing LC-OCT alongside other diagnostic modalities, clinicians can achieve a more accurate diagnosis, ensuring that appropriate management strategies are implemented for both benign and malignant skin lesions. Our LC-OCT findings align with well-established histopathological characteristics of hidrocystomas, which are cystic spaces in the dermis typically lined by a dual-layered epithelium [21,30] (Figure 3).

**Figure 4:**
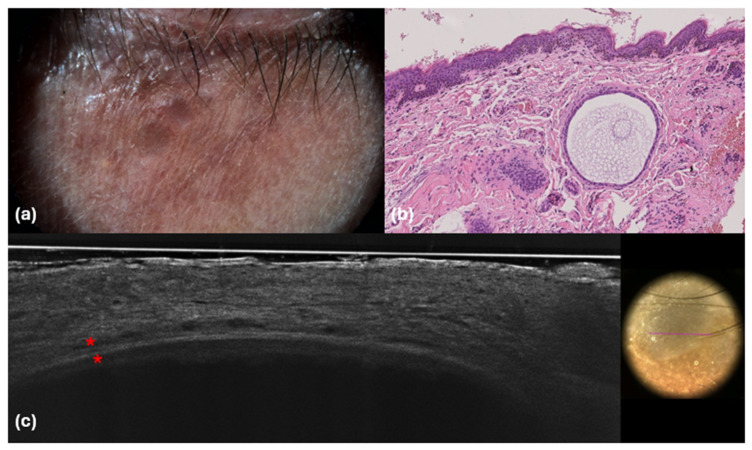
Dermoscopic (**a**), histological (**b**) and LC-OCT (**c**) images. (**a**) Purple-bluish color; (**b**) dermal cystic lesion; (**c**) hyporeflective cupuliform area well defined by a bright and thick upper contour with two bright layers of cells (red asterisks). Hematoxylin and Eosin; original magnification ×20.

Indeed, within the thick hyperreflective contour visible in LC-OCT, it was often possible to identify two bright layers of cells (Figure 4).

However, the deeper margin of the cystic lacunae was not visible in any image because it was situated too deep.

### 4.3. RCM

**Figure 5:**
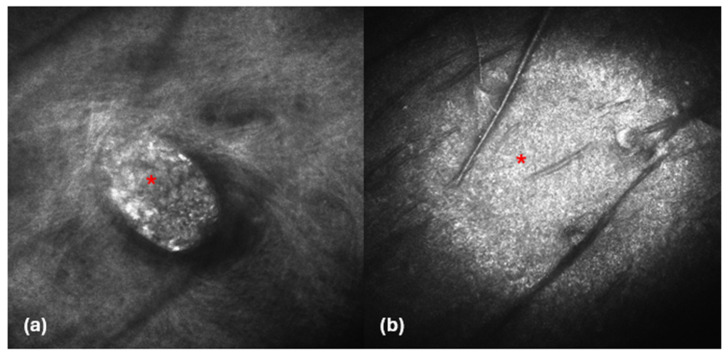
RCM images (**a**,**b**). Hyperreflective oval structure probably corresponding to calcification (red asterisks).

The role of RCM in diagnosing hidrocystomas was thoroughly explored in a recent study conducted by Cinotti et al. [16]. In their findings, hidrocystomas were characterized as dark areas surrounded by a well-defined two-layer epithelium, with no detectable epidermal architectural anomalies present. This absence of typical epidermal disruptions is significant, as it suggests that RCM can effectively identify the structural integrity of the epidermis in these benign lesions. Also, the study emphasized that distinct features commonly associated with basal cell carcinoma (BCC) were entirely absent in all examined cases, reinforcing the non-malignant nature of hidrocystomas [16]. In addition, other studies [17,18,19] have corroborated these findings, noting a normal epidermal structure adjacent to round dark formations that corresponded to normal adnexal structures. Hyporeflective lacunae were also observed, indicating the presence of cystic structures within the dermis. However, in our study, RCM was performed on two patients and no definitive signs of hidrocystomas were identified, which may be attributed to the deeper anatomical location of the lesions that could limit the visibility of RCM. Despite the lack of typical findings, both the epidermis and dermis appeared normal upon examination. In one particular case, a hyperreflective oval structure was noted, which we hypothesized could correspond to a calcification that was not visible through the LC-OCT imaging modality (Figure 5).

### 4.4. UHFUS

**Figure 6:**
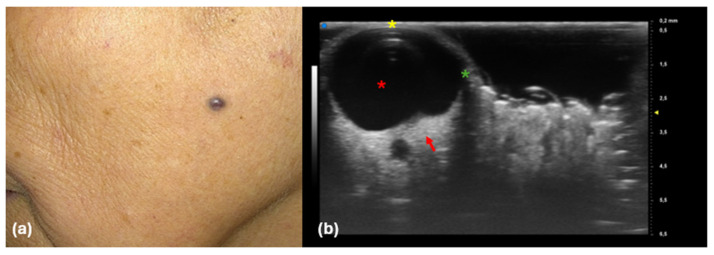
Clinical (**a**) and UHFUS (**b**) images. (**a**) Purple-bluish papule on the cheek; (**b**) anechoic round areas within the dermis (red asterisk), featuring a thick superficial roof (yellow asterisk), lateral hypoechoic bands (green asterisk), and a hyperechoic deep margin (red arrow), likely due to the fluid content within these structures.

Finally, UHFUS was performed in two cases, yielding significant insights into the structural characteristics of hidrocystomas. The ultrasound examination revealed either single or multiple anechoic round areas situated within the dermis. These anechoic zones were particularly notable for their thick superficial roof, which likely indicates the presence of fluid, providing a clear distinction between cystic and surrounding tissue. Additionally, lateral hypoechoic bands were observed, suggesting changes in the echogenicity of the adjacent dermis, possibly due to variations in tissue composition or inflammation. A hyperechoic deep margin was also noted, which is commonly attributed to the fluid content within these cystic structures, enhancing their visibility on ultrasound (Figure 6).

Although there is limited research regarding UHFUS and hidrocystomas, our findings align with those of Wang YK et al., who noted lateral acoustic shadows and increased posterior echogenicity [24]. Interestingly, UHFUS effectively identified the cystic characteristics of these lesions, distinguishing them from non-cystic conditions like BCC, which are typically less anechoic, round, and less well defined [32]. Additionally, the ultrasound results corroborated the LC-OCT images, with UHFUS providing insight into deeper margins due to its superior depth penetration.

This capability of UHFUS to highlight cystic features is particularly important in clinical practice, as it provides dermatologists with a non-invasive method to better visualize and assess lesions before considering more invasive procedures. Furthermore, the ultrasound findings not only complemented the observations made using LC-OCT but also provided deeper insights into the anatomical margins of the lesions. The superior depth penetration of UHFUS allowed for a more comprehensive evaluation of the hidrocystomas, enhancing our understanding of their anatomical features and supporting more accurate diagnostic conclusions. This holistic approach underscores the importance of integrating multiple imaging modalities in dermatological practice, ultimately improving patient outcomes through precise diagnosis and tailored management strategies.

Diagnosing hidrocystomas in clinical practice can be challenging. Clinical presentation and dermoscopy alone can be insufficient for a definitive diagnosis. Therefore, additional non-invasive imaging techniques, such as LC-OCT and UHFUS, can improve diagnostic accuracy by highlighting benign features and facilitating the early exclusion of BCC, which can help reassure the patient. Given the facial location of hidrocystomas, these real-time imaging modalities can expedite the diagnostic process and help avoid unnecessary skin biopsies that could have functional and esthetic repercussions. However, several limitations should be noted. The primary limitation is the small sample size, which is largely attributable to the rarity of the condition. Another significant constraint is the retrospective design, which may introduce observational biases. Additionally, considering the benign nature of these lesions, only three underwent histopathological examination.

## 5. Conclusions

In conclusion, we have described the dermoscopic, RCM, LC-OCT, and UHFUS characteristics of hidrocystomas, highlighting the potential of these techniques to enhance diagnostic confidence and prevent unnecessary surgical excision. Non-invasive imaging methods may facilitate differentiation between hidrocystomas and malignant tumors like BCC and assist in treatment monitoring. Particularly, when clinical and dermoscopic examinations are inconclusive, LC-OCT and UHFUS can reveal specific benign features, providing crucial support for clinicians. Nevertheless, further research is needed in this area, particularly prospective controlled studies with larger sample sizes.

## Figures and Tables

**Table 1:** Distribution of clinicopathological, dermatoscopic, and LC-OCT features of hidrocystomas.

	Hidrocystomas
**Clinical features, *n* (%)**	
Papules	7 (100)
Translucency	7 (100)
Purple-bluish color	5 (71)
**Dermoscopic features, *n* (%)**	
Purple-bluish color	5 (71)
White pale halo	5 (71)
Whitish globules	4 (57)
Chrysalis	3 (43)
Arborizing vessels	4 (57)
Linear vessels	2 (28)
**LC-OCT features, *n* (%)**	
Hyporeflective cupoliform round structure in the dermis	5 (100)
Bright and thick contour	5 (100)
Hyporeflective content	4 (80)
Hyperreflective content	1 (20)
**UHFUS features, *n* (%)**	
Anechoic round areas within the dermis	2 (100)
Thick superficial roof	2 (100)
Lateral hypoechoic bands	2 (100)
Hyperechoic deep margin	2 (100)

**Table 2:** LC-OCT: main features of BCC and hidrocystomas [31].

BCC	Hidrocystoma
Lobule	Hyporeflective cupoliform area
Structure of variable shape, size and location within the dermis, with a gray core surrounded by a darker rim. The Millefeuille pattern corresponds to a grey, laminated LC-OCT pattern within the lobule, horizontally oriented. Clefting represents a dark rim surrounding the core of the lobule, while a bright rim (brighter than the stroma) often surrounds the lobule.	Well-defined area, delimited by a bright and thick upper contour, corresponding to the top of the hidrocystoma. The content of the lesion is filled with a completely homogeneous hyporeflective material. A thick hyperreflective contour surrounds the cupoliform structure.
Vascular structures	Vascular structures
Well-defined, hyporeflective vessels of various shapes/size, located within the dermis and especially next to lobules.	Dilated vessels situated above the cupoliform area, not consistently visible.
Epidermal changes	Epidermal changes
Parakeratosis, disorganized epidermis.	Not detected.
Bright cells within epidermis/lobules	Bright cells within epidermis/lobules
Hyperreflective structures within the epidermis and/or the lobules.	Not detected.

## Data Availability

The data presented in this study are available upon request from the corresponding author.
